# Improved Electrochemical Detection of Zinc Ions Using Electrode Modified with Electrochemically Reduced Graphene Oxide

**DOI:** 10.3390/ma9010031

**Published:** 2016-01-07

**Authors:** Jiri Kudr, Lukas Richtera, Lukas Nejdl, Kledi Xhaxhiu, Petr Vitek, Branislav Rutkay-Nedecky, David Hynek, Pavel Kopel, Vojtech Adam, Rene Kizek

**Affiliations:** 1 Department of Chemistry and Biochemistry, Mendel University in Brno, Zemedelska 1, Brno CZ-613 00, Czech Republic; george.kudr@centrum.cz (J.K.); oliver@centrum.cz (L.R.); lukasnejdl@gmail.com (L.N.); kledi.xhaxhiu@fshn.edu.al (K.X.); brano.ruttkay@seznam.cz (B.R.-N.); d.hynek@email.cz (D.H.); paulko@centrum.cz (P.K.); vojtech.adam@mendelu.cz (V.A.); 2 Central European Institute of Technology, Brno University of Technology, Technicka 3058/10, Brno CZ-616 00, Czech Republic; 3 Global Change Research Institute, The Czech Academy of Sciences, v.v.i., Bělidla 4a, Brno CZ-603 00, Czech Republic; vitek.p@czechglobe.cz; 4 Department of Biomedical and Environmental Analysis, Wroclaw Medical University, Borowska 211, Wrocław PL-50 556, Poland

**Keywords:** carbon, cyclic voltammetry, electrochemical impedance spectroscopy, electrochemistry, graphene oxide, heavy metal detection, reduced graphene oxide

## Abstract

Increasing urbanization and industrialization lead to the release of metals into the biosphere, which has become a serious issue for public health. In this paper, the direct electrochemical reduction of zinc ions is studied using electrochemically reduced graphene oxide (ERGO) modified glassy carbon electrode (GCE). The graphene oxide (GO) was fabricated using modified Hummers method and was electrochemically reduced on the surface of GCE by performing cyclic voltammograms from 0 to −1.5 V. The modification was optimized and properties of electrodes were determined using electrochemical impedance spectroscopy (EIS) and cyclic voltammetry (CV). The determination of Zn(II) was performed using differential pulse voltammetry technique, platinum wire as a counter electrode, and Ag/AgCl/3 M KCl reference electrode. Compared to the bare GCE the modified GCE/ERGO shows three times better electrocatalytic activity towards zinc ions, with an increase of reduction current along with a negative shift of reduction potential. Using GCE/ERGO detection limit 5 ng·mL^−1^ was obtained.

## 1. Introduction

Heavy metal pollution has become a major concern all over the world. Anthropogenic processes like urbanization and industrialization have led to their release from Earth’s crust and their accumulation in the biosphere. The long-term monitoring of heavy metal pollution is the only way to meet the legislative demands and decrease pressure on the environment. However, most heavy metals like lead or cadmium are toxic even at low concentrations, others, which belong to a group of essential micronutrients, pose health risks in high supplementation only, but their monitoring is also needed [1,2,3]. Among essential micronutrients, zinc(II) plays one of the most important role. Zinc analysis is appealing not only from the environmental point of view but also from the biochemical one. Zinc(II) ions play an important role in cell replication and nucleic acid metabolism, and its deficiency is connected with some pathological processes like retarded growth and immunity dysfunction [4]. As was shown recently, the enhanced zinc intake by drinking water in the case of mice caused zinc deficiency in the hippocampus, associated with memory deficit and decreased expression levels of learning and memory related receptors [5]. Zinc has these important roles and effects mainly as a co-factor of numerous proteins, therefore it is not surprising that metallomics and proteomics of zinc-containing proteins are emerging fields of science [6,7,8]. From these, metallothioneins are highlighted as maintainers and transporters of these proteins and their importance in zinc metabolism belongs to the interest of numerous researchers [9,10,11,12,13,14,15,16,17,18].

Atomic absorption spectrometry (AAS) and inductively coupled plasma mass spectrometry (ICP-MS) represent a gold standard in detection of trace heavy metals concentrations. Nevertheless, they require expensive instrumentation, experienced operators, and the analyses are time-consuming. On the contrary, electrochemistry offers superior features like portability, easy use, low price, miniaturization, and high sensitivity [19,20,21,22,23]. The great advantage of electroanalysis is also the possibility of electrode surface modification [24].

Mercury electrodes have been widely used in trace heavy metal analysis for decades; however, they do not correspond with current trends including miniaturization. Whereas material sciences are a rapidly developing field of science, several micro to nanosized materials like liquid metals/metal oxides in order to improve electrode properties are attracting the attention of analysts [25,26,27,28]. Graphene, theoretically perfect two-dimensional (one-atom-thick) material, is the ideal choice for electrochemistry since it possesses unusual electronic conductivity and high surface area [29]. However, it is worth noting that a one-atom-thick, defect-less graphene monolayer is difficult to prepare and standard graphene materials are far from perfectly structured, and therefore more often reduced graphene oxide (rGO) is used. The procedure of GO reduction influences subsequent rGO properties. Electrodes modified with rGO obtained using constant potential chemical and thermal reduction was previously compared [30]. From electrochemical methods for GO reduction cyclic voltammetry was also used [31]. Electrodes modified with rGO are not only desirable for just electroanalytical chemistry, but also for the removal of organic pollutants from wastewaters [32]. Various methods have been used to prepare electrodes modified with GO [33,34,35,36]. Among others, electrodeposition of GO or rGO attracted interest due to its efficiency, ease of use, and rapid procedure [37]. Potentiostatic methods and cyclic voltammetry (CV) were shown to be suitable tool for electrodeposition of these materials on electrodes [38,39]. Recently, pulse potential method based on changing of anodic deposition and cathodic reduction periods was developed too [40]. Moreover, an electrode surface modification with biomolecules or graphene-like nanomaterials can significantly improve detection sensitivity and selectivity [41,42,43].

In this work the GO film on glassy carbon electrode (GCE) was fabricated by the potentiostatic deposition of GO. Electrochemically deposited GO was subsequently subjected to electrochemical reduction to produce electrochemically reduced graphene oxide (ERGO) using CV. The properties of this modified electrode were compared with standard bare GCE using CV and electrochemical impedance spectroscopy (EIS). [Fe(CN)_6_]^3−^/[Fe(CN)_6_]^4−^ was used as a redox probe for electrode characterization and the performance of GCE/ERGO on detection of Zn(II) using differential pulse voltammetry (DPV) was examined.

## 2. Results and Discussion

### 2.1. Preparation of GCE/ERGO

Since the discovery of graphene, it has been attracting great attention due to its high conductivity and surface to volume ratio [44]. However, from an electrochemical point of view graphene suffers from a limited number of hydrophilic moieties and electroactive sites [45]. GO with randomly distributed oxygen groups benefits from structural similarity with graphene, nevertheless structure breaks cause the decrease of conductivity [46]. Partially reduced GO represents an intermediate between ideal graphene structure and GO, whereas the amount of electroactive surface and reactive functionalities (epoxy, hydroxyl, carboxyl) are balanced.

The common method to fabricate rGO is exfoliation of graphite to produce GO followed by thermal or chemical reduction [47]. ERGO represents an alternative since no expensive equipment or use of toxic compounds is needed during its fabrication. Several procedures have been introduced to cover electrodes with a GO or GO/ERGO layer [34,38,48,49]. Direct electrodeposition from solution or drop-casting of GO or rGO on the surface of electrode can be used. If GO is used as a source material for electrode modification, deposition is followed by electrochemical reduction of GO to prepare ERGO. Previously, CV, potentiostatic or pulsed methods (several cycles of deposition in positive potential followed by GO reduction in negative potential) were used in order to cover the electrode with ERGO [50,51].

**Figure 1:**
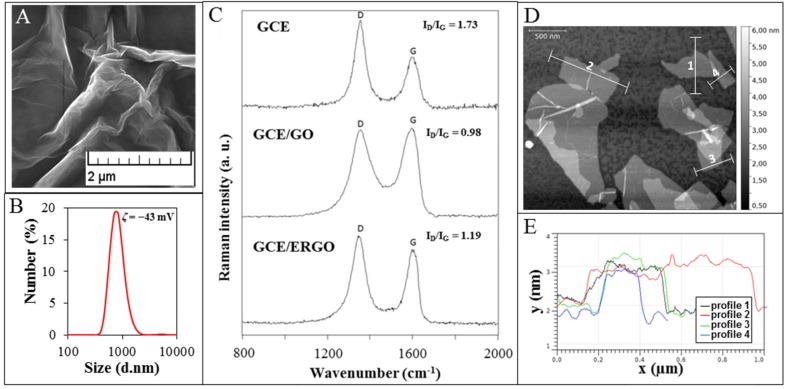
(**A**) Micrograph of GO used to modify GCE obtained by SEM; (**B**) GO size distribution including zetapotential; (**C**) Raman spectra of GCE, GCE modified with GO and GCE modified with ERGO; (**D**) AFM image of GO and (**E**) the height profiles along lines displayed in AFM image.

Here, GO was prepared according to the simplified Hummer’s method (Figure 1A). It was revealed that our GO sample contains particles with hydrodynamic diameter of 848 ± 290 nm (Figure 1B). Negative charge of GO was confirmed using measurement of zetapotential (ζ = −43 mV), which enables to deposit GO particles on the electrode using application of positive potential on it. This value also suggests that GO particles possess good stability in colloid phase.

**Figure 2:**
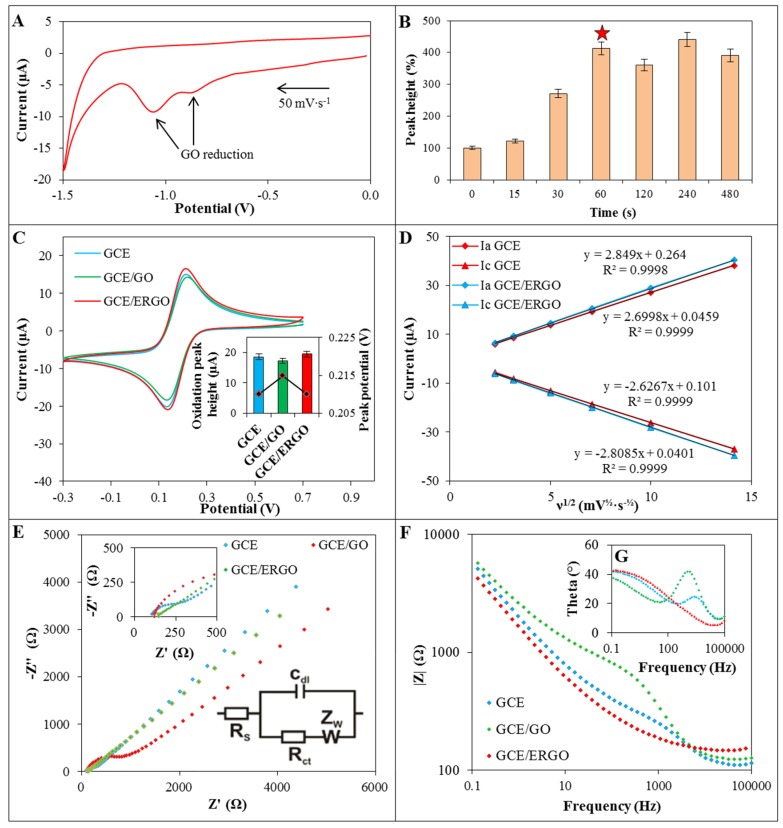
(**A**) The CV (0 − (−1.5) V) of GCE/GO in acetate buffer; (**B**) Dependence of Zn(II) reduction signal obtained using GCE/ERGO on deposition time of GO (0.5 mg·mL^−1^) on the electrode (deposition time selected as optimal is marked with star); (**C**) CV of 2 mM [Fe(CN)_6_]^3−^/[Fe(CN)_6_]^4−^ in 0.1 M KCl (50 mV∙s^−1^) recorded on bare GCE (blue line), GCE/GO (red line) and GCE/ERGO (green line) and corresponding peak current levels; (**D**) The dependence of [Fe(CN)_6_]^3−^/[Fe(CN)_6_]^4−^ anodic (Ia) and cathodic (Ic) peak heights on the square root of scan rate; (**E**) Nyquist plot, detail of nyquist plot high frequency region and equivalent circuit used for data evaluation in insets; (**F**) Bode modulus plot of bare GCE (blue line), GCE/GO (red line) and GCE/ERGO (green line); and (**G**) corresponding Bode phase diagram (same colours as previous figure).

For the fabrication of GCE/ERGO, the constant potential +1.0 V *vs.* Ag/AgCl/3 M KCl reference electrode were applied to GCE in a previously sonicated water solution of GO (0.5 mg·mL^−1^). Due to the presence of oxygen-containing functionalities negatively charged GO is electrostatically attracted to positively charge electrode. Subsequently, the working electrode was gently rinsed with water, transferred to acetate buffer and five CV cycles (from 0 to −1.50 V) were performed [52]. The irreversible reduction signals at −1.05 V and −0.85 V were observed in first cycle and completely disappeared in subsequent cycles (Figure 2A). It was shown previously that reduction of GO provides peak around −1.10 V [8,45,51]. Nevertheless different oxygen-containing moieties can be presented within GO, which can result in different reduction signals. The deposition (0–480 s) time of GO on electrode was optimized using detection of 20 µmol·L^−1^ Zn(II) signal and the deposition for 60 s was found as an optimal (Figure 2B). It was shown that although deposition of GO increased reduction signal of zinc slightly for 15 s, deposition of GO increased signal nearly three-fold for 30 s when compared with bare GCE cathodized for precise time in water. The deposition time 60 s was able to sufficiently modify the surface of GCE with GO, and the increase of deposition time did not result in the increase of detection signal.

Further, we analyzed the surface of the modified electrode by Raman spectroscopy. The D and G Raman bands were detected at 1355 cm^−1^ and 1595 cm^−1^ for GO and 1348 cm^−1^ and 1600 cm^−1^ for ERGO, both deposited on GCE. The Raman intensity ratio of the D and G bands (*I_D_/I_G_*) is increased in the case of GCE/ERGO (1.19) compared to GCE/GO (0.98) (Figure 1C), which is in accordance with literature [53,54]. It is attributed to the modification of the GO structure by reduction resulting in removal of functional groups and creation of defects between the *sp*^2^ domains [55]. Change of full width at half maximum (FWHM) was observed from 115 cm^−1^ for GO towards 78 cm^−1^ in the case of ERGO for D band. The value for ERGO points at high disorder with low distances between defects [55]. The image of GO obtained using AFM suggests that GO is presented within sample in sheet-like shapes (Figure 1D). The thickness of GO, deduced from the height profile of AFM image, is about 1 nm, which is comparable to GO monolayer thickness published previously [56,57].

### 2.2. Characterization of GCE/ERGO

In order to characterize GCE/ERGO, cyclic voltammograms of equimolar 2 mmol·L^−1^ [Fe(CN)_6_]^3−^/[Fe(CN)_6_]^4−^ as a redox probe was measured using bare GCE and GCE/GO and compared with the record measured using GCE/ERGO. As it is shown in Figure 2C, deposition of GO on GCE reduces the peak current by 5%. On the contrary, GCE/ERGO exhibited better detection properties by 10% (inset in Figure 2C). Based on these data, the Randles-Sevcik equation (Equation (1)) was used to calculate the electroactive surface area of bare GCE and subsequently compare it with GCE/ERGO. The areas of 6.4 mm^2^ and 7.0 mm^2^ were acquired, respectively, which means increase for about 9.4% and confirm successful deposition and reduction of GO. The values of reduction and oxidation peaks of [Fe(CN)_6_]^3−^/[Fe(CN)_6_]^4−^ were plotted against the square root of scan rates (Figure 2D). The linear dependence revealed diffusion controlled processes for both GCE and GCE/ERGO and slightly improved sensitivity of detection in case of GCE/ERGO. These results were also confirmed by EIS (Figure 2E).

The Randles circuit was used as an equivalent circuit for fitting the EIS data. It consisted of solution resistance Rs, charge transfer resistance Rct, double layer capacitance Cdl and Warburg impedance ZW (inset in Figure 2E). Nyquist diagram showed in case of bare GCE depressed semicircle with charge transfer resistance 2.1 kΩ·cm^−1^. After deposition of GO on GCE, charge transfer resistance increased four-fold to 8.5 kΩ·cm^−1^. Very small depressed semicircle was observed in the case of GCE/ERGO, where Rct decreased to 0.6 kΩ·cm^−1^ (32% of GCE Rct). Significantly lower charge transfer resistance of GCE/ERGO in comparison with GCE/GO was previously reported [58]. In Bode diagram the frequency dependence on absolute magnitudes of impedance modulus |Z| was plotted (Figure 2F). The peaks of Bode phase diagram in case of GCE and GCE/GO (1–3 kHz) suggests that charge transfer resistance takes place in the electrode/electrolyte interface. Phase peak of Bode plot of GCE/ERGO disappeared at higher frequencies as a result of high electron transfer, where charge transfer resistance decreased (Figure 2G).

### 2.3. Detection of Zn(II)

**Figure 3:**
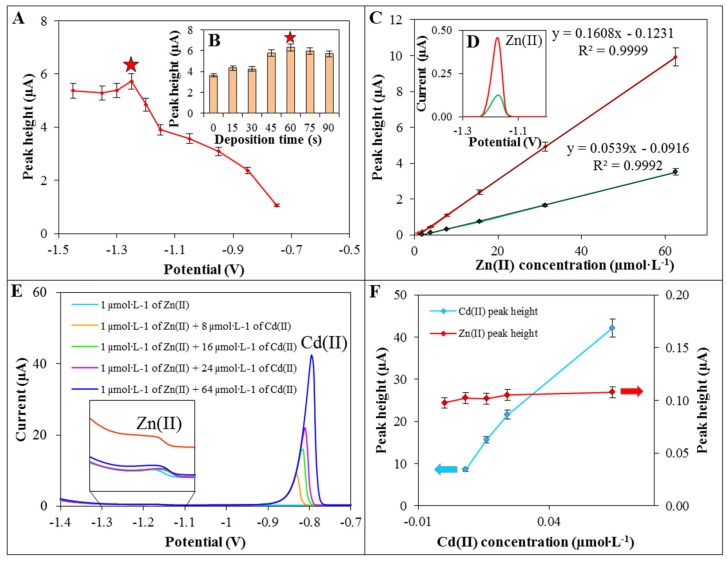
Dependence of Zn(II) reduction signal on deposition potential (**A**) and deposition time (**B**) of Zn(II) (35 µmol·L^−1^) on GCE/ERGO (parameters marked with star was selected as an optimal); (**C**) Dependence of electrochemical signal on Zn(II) concentration (1.0–62.5 µmol·L^−1^) and comparison of DPV reduction signals of Zn(II) (4 µmol·L^−1^) (**D**) obtained using GCE/ERGO (red line) and bare GCE (blue line); (**E**) DPV voltammograms ((−1.40) − (−0.70) V) and comparison of Zn(II) and Cd(II) peak heights (**F**) of Zn(II) solution (1 µmol·L^−1^) with different concentrations of Cd(II) (0–64 µmol·L^−1^). Comparison of 10 µmol·L^−1^ Zn(II) electrochemical signal in acetate buffer with added 50 µmol·L^−1^ K(I), Ca(II) and Mg(II) in inset.

The detection of Zn(II) was performed using DPV. Firstly, deposition potentials ((−1.45) − (−0.65) V) of Zn(II) on the surface of GCE/ERGO was optimized. As it can be seen in Figure 3A, the obtained Zn(II) signal increased from potential −0.65 to −1.25 V. At potential −1.25 V the Zn(II) signal reached its higher value and was choose as an optimal. As the next step, deposition time (0–90 s) of Zn(II) was optimized (Figure 3B). It was revealed that the signal increased by 73% using deposition time 60 s and deposition potential −1.25 V in comparison with deposition time 0 s. After this optimization, different concentrations of Zn(II) were measured using GCE/ERGO and calibration curve was determined (Figure 3C). It exhibited linear section between 1.0 µmol·L^−1^ and 62.5 µmol·L^−1^ and other analytical parameters of detection are displayed in Table 1. The modification of GCE with ERGO improved the detection of zinc ions (35 µmol·L^−1^) four-fold in comparison with bare GCE and slightly shifted peak potential from −1.18 V to −1.2 V (Figure 3D). Using GCE/ERGO, we obtained limit of detection (LOD) 0.1 µmol·L^−1^ Zn(II) (~5 ng·mL^−1^).

**Table 1:** Analytical parameters of electrochemical detection of Zn(II).

Substance	Working Electrode	Regression Equation	Linear Dynamic Range (µmol·L^−1^)	R^2 a^	LOD ^b^ (µmol·L^−1^)	LOQ ^c^ (µmol·L^−1^)	RSD (%)
Zn(II)	GCE/ERGO	*y* = 0.1608*x* − 0.1231	62.5 – 1.0	0.9999	0.1	0.4	4.8
Zn(II)	GCE	*y* = 0.0539*x* − 0.0916	500.0 – 2.0	0.9992	0.5	2.0	5.2

^a^ Regression coefficient; ^b^ LOD (*S*/*N* = 3); ^c^ LOQ (*S*/*N* = 10).

As the final step, the effect of interference with Zn(II) detections was examined. We chose Cd(II) since it is quite often presented in environmental samples and may affect Zn(II) detection [41,59]. Zn(II) solutions (1 µmol·L^−1^) with different concentrations of Cd(II) (0–64 µmol·L^−1^) were measured and the peak heights of Zn(II) (potential −1.15 V) were compared (Figure 3E). As it can be seen, peak of Cd(II) (about potential −0.80 V) is well separated from Zn(II) peak and did not significantly affect Zn(II) peak heights even at a 64-times higher concentration (Figure 3F). In addition, other monovalent and bivalent ions were tested as a possible interference in real sample. To Zn(II) solution five-times higher concentrations of K(I), Ca(II), and Mg(II) ions were added and their effects on Zn(II) were evaluated. All Zn(II) analysis presented here were performed in the acetate buffer where Na(I) ions were present in high concentration. Other tested ions showed no apparent interference in Zn(II) detection (Figure 3F inset).

## 3. Experimental Section

### 3.1. Chemicals and Material

ACS purity (*i.e.*, chemicals meet the specifications of the American Chemical Society) sodium acetate trihydrate, acetic acid, zinc nitrate, potassium hexacyanoferrate(III), potassium hexacyanoferrate(II) trihydrate, potassium chloride, water, and other chemicals were purchased from Sigma-Aldrich (St. Louis, MO, USA) unless noted otherwise.

### 3.2. Preparation of GO

GO was synthesized using chemical oxidation of graphite flakes (5.0 g, Sigma-Aldrich, and 100 mesh, ≥75% min) in a mixture of concentrated H_2_SO_4_ (670 mL, ACS reagent 95.0%–98.0%) and 30.0 g KMnO_4_ (>99%) according to the simplified Hummer’s method [60]. The reaction mixture was stirred vigorously. After four days, the oxidation of graphite was terminated by slow adding of H_2_O_2_ solution (250 mL, 30 wt % in H_2_O) and the colour of the mixture turned to bright yellow, indicating high oxidation level of graphite. Formed graphite oxide was washed three times with 1 M of HCl and washed with water several times (total volume used 12 L) until constant pH value (4–5) was achieved using a simple decantation. Then, it was possible to centrifuge this solution. During the washing process with deionized water, exfoliation of graphite oxide led to the thickening of solution and formation of a stable colloid of GO.

### 3.3. Glassy Carbon Electrode Modification with Graphene

GCE was mechanically polished by the 1.0 µm and 0.3 µm alumina suspension (CH Instruments, Austin, TX, USA) on polishing cloth to produce mirror-like surface. Then, the electrode was sonicated for 3 min in distilled water (25 °C) and acetone successively in the Sonorex digital 10 P ultrasonic bath (Bandelin, Berlin, Germany). As prepared, the electrode was rinsed with water solution of GO (0.5 mg·mL^−1^) and potential +1.0 V was applied on working electrode *vs.* Ag/AgCl/3 M KCl. The deposited film of GO was reduced by performing CV from 0.0 V to −1.5 V in acetate buffer (0.2 M, pH = 5) to produce ERGO.

### 3.4. Instrumentation

Determination of Zn(II) and [Fe(CN)_6_]^3−^/[Fe(CN)_6_]^4−^ by DPV and CV respectively was performed using PGSTAT302N (Metrohm, Herisau, Switzerland) using a three electrode system. A 3 mm diameter GCE (CH Instruments, Austin, TX, USA) was employed as the working electrode. An Ag/AgCl/3 M KCl electrode was used as the reference and platinum wire served as auxiliary. For data processing NOVA 1.8 (Metrohm, Herisau, Switzerland) was employed. Acetate buffer (0.2 mol·L^−1^ CH_3_COONa and CH_3_COOH, pH = 5) and 0.2 mol·L^−1^ KCl were used as a supporting electrolyte in cases of Zn(II) and [Fe(CN)_6_]^3−^/[Fe(CN)_6_]^4−^ determination, respectively.

The parameters of the measurement by DPV were as it follows: initial potential −1.3 V, end potential −1.0 V, deposition time 60 s, time interval 0.03 s, step potential 5 mV, scan rate 50 mV·s^−1^. Parameters of the measurement by CV were as it follows: initial potential of −0.3 V, upper vertex potential 0.7 V, lower vertex potential −0.3 V, step potential 2.4 mV, scan rate 50 mV·s^−1^. All measurements were carried out at 25 ± 1 °C.

The value of formal potential of [Fe(CN)_6_]^3−^ in 0.1 mol·L^−1^ KCl was 0.25 V and we also adopted it at impedance measurements. Impedance spectra were measured from 0.1 Hz to 10^5^ Hz with alternating current (AC) amplitude of 10 mV. PGSTAT302N (Metrohm, Herisau, Switzerland) was used for impedance measurements with the same three electrode system as mentioned previously. Individual elements of equivalent circuit were calculated using NOVA 1.8 (Metrohm, Herisau, Switzerland).

### 3.5. The Electroactive Surface Determination

In order to determine electroactive area of GCE and to compare it with GCE/ERGO, cyclic voltammograms of 2 mM [Fe(CN)_6_]^3−^/[Fe(CN)_6_]^4−^ in 0.1 M KCl were recorded using the aforementioned electrodes. Electroactive surface was calculated according to Randles-Sevcik equation:

(1) $$I_{p}=2.69\cdot 10^{5}A\cdot D^{\frac{1}{2}}n^{\frac{3}{2}}\nu^{\frac{1}{2}}C$$

where *I_p_* is anodic current peak (A), *A* is the electroactive area (cm^2^), *D* is the diffusion coefficient of [Fe(CN)_6_]^4−^ in solution (6.1 × 10^−6^ cm^2^·s^−1^ was taken according to Prathish *et al.* [61]), *n* is the number of electrons transferred in half-reaction (1 in case of [Fe(CN)_6_]^4−^), ν is scan rate (0.05 V∙s^−1^ was chosen) and *C* is [Fe(CN)_6_]^4−^ concentration (mol∙L^−1^).

### 3.6. Scanning Electron Microscopy (SEM)

Structure of carbon materials were characterized by SEM. For documentation of the structure, a MIRA3 LMU (Tescan, Brno, Czech Republic) was used. The SEM was fitted with In-Beam SE detector. For automated acquisition of selected areas a TESCAN proprietary software tool called Image Snapper was used. The software enabled automatic acquisition of selected areas with defined resolution. An accelerating voltage of 15 kV and beam currents about 1 nA gave satisfactory results.

### 3.7. Dynamic Light Scattering (DLS)

Average particle size, size distribution, and particle zetapotential were determined by dynamic light scattering method by Zetasizer Nano-ZS (Malvern Instruments Ltd., Worchestershire, UK) with a scattering angle θ = 173°. Samples were measured in water solution.

### 3.8. Raman Spectroscopy

All carbonaceous materials, bare GCE, GO, and ERGO deposited on GCE were characterized by Raman spectroscopy. Measurements were performed on a Renishaw *InVia* Reflex Raman microspectrometer equipped by the 514.5 nm line of an argon laser for excitation. A Leica microscope equipped with a standard 50× objective were used. The laser power was set to 1–2 mW at source to obtain an optimal Raman signal and simultaneously avoid any thermal alteration of the sample. Scans of 5–8 s were accumulated 10 times. Resulting spectra were baseline-corrected in GRAMS/AI 9.1.

### 3.9. Interference Measurement

Zn(NO_3_)_2_ was mixed with KCl, CaCl_2_, and MgCl_2_ to obtain a final concentration of 10 µmol·L^−1^ Zn(II) and 50 µmol·L^−1^ K(I), Ca(II) and Mg(II), respectively, in acetate buffer. Obtained Zn(II) reduction signals were compared with a signal of 10 µmol·L^−1^ Zn(II) in acetate buffer.

### 3.10. Atomic Force Microscopy Measurement

#### 3.10.1. GO Immobilization

GO was immobilized on freshly cleaved mica surfaces grade V-1 (Structure Probe/SPI Supplies, West Chester, PA, USA). The mica surface was first modified by silanization in vapours of *N*-aminopropyldimethylethoxysilane (APDMES) with catalysis of *N*,*N*-diisopropylethylamine (DiPEA, both from Sigma Aldrich). Fifty microliters of GO stock solution 10-times diluted in double distilled water was subsequently transferred onto the modified mica surface and left to incubate for 15 min in a wet chamber under laboratory temperature. Then the surface was carefully washed with double distilled water and left to dry in desiccators for another 30 min (10 Pa vacuum).

#### 3.10.2. Visualization of GO

The AFM images of GO fixed on mica sheets were taken by Bruker Dimension FastScan atomic force microscope (Bruker Nano Surface, Santa Barbara, CA, USA) operated in tapping mode. Basic parameters of the visualization process were as follows: set point value 3.5 nm, iGain 0.8, PGain 5.5, piezo *Z* scale range 500 nm. All images were collected under ambient conditions at 38% relative humidity and 22.5 °C with a scanning raster rate of 2.0 Hz. Silicon nitride triangular cantilevers “FastScan A” (Bruker Nano Surface) characterized by spring constant of 17 N/m and resonant frequency of 1397 kHz equipped with tetrahedral silicon tip with nominal tip radius 5 nm were used for imaging.

Gwyddion software [62] version 2.43 was used for AFM data post processing and for graphical output.

## 4. Conclusions

Modifications of electrode surface, where redox processes in electrochemical measurements take place, are promising techniques to improve detection sensitivity. Nanomaterials and different carbon materials among others are nowadays frequently used to meet this goal. As it was evident from our measurements, graphene modification of working electrodes is an easy way to enhance electrode properties. GO was electrodeposited from solution on GCE at constant positive potential and subsequently electrochemically reduced using cyclic voltammetry measurement at negative potentials. Proved by Raman and electrochemical impedance spectroscopy, successful modification of the electrode resulted in an increase of electroactive surface area by 9.4% compared with bare GCE. We found that GCE/ERGO possesses three-fold higher sensitivity for zinc ions in comparison with bare GCE. Acceptable selectivity towards interfering ions, such as K(I), Cd(II), Mg(II), and Ca(II) was achieved.
